# Where Shall We Send Our Consumptive Patients?

**Published:** 1871-11

**Authors:** 


### Where shall we send our Consumptive Patients?

Every day the enquiry is made as to what locality on this coast is best adapted for a sanitarium—a place for convalescents, invalids, and consumptives. That no one locality will suit all cases is a palpable truth. An individual threatened with phthisis might find health in the mountains, during the summer, where he might even "camp out" with benefit; or a journey in the saddle, or a sea voyage might restore his health. The same may be said of persons affected with dyspepsia and similar disorders. But delicate females, and consumptive patients in more advanced stages of disease, must seek relief elsewhere. The summer winds of the bay and ocean climate are too chill; the interior is too hot and debilitating. There is a middle region, a narrow district skirting the bay, enjoying a medium climate. It embraces portions of the counties of Marin, Sonoma, Napa, Contra Costa, Alameda, Santa Clara, and San Mateo. But this is so often a battle-ground between the two climates, in which wind and mist on the one hand, and a broiling sun on the other, triumph alternately, that it does not supply the need. In the southern counties of the State is a range of territory some miles inland from the coast, which enjoys a more equable climate, both in summer and in winter. Los Angelos and San Diego are the two most attractive localities in this range, and the inhabitants of each place think their town the most salubrious spot on the globe. San Diego is more exempt from summer heat than Los Angelos, and being nearer the ocean, has a more equable winter temperature. The inhabitants have secured a large stock of thermometers and pluviometers, and have become xealous meteorologists, and determined to demonstrate the unparalleled sanitary virtues of their growing burgh. Thus far San Diego has the lead in the race, and presents the strongest inducements to valetudinarians.—*Pacific Medical and Surgical Journal.*

